# The impact of the pandemic on the perception of stress and danger, and the adjustment of psychiatric and general medical staff of German military hospitals

**DOI:** 10.3389/fpsyt.2023.1141052

**Published:** 2023-05-16

**Authors:** Gerd-Dieter Willmund, Johannes Müller, Niels Schneegans, Helge Höllmer, Ulrich Wesemann, Peter Lutz Zimmermann, Christian Helms

**Affiliations:** ^1^Bundeswehr Centre for Military Mental Health, Bundeswehrkrankenhaus Berlin, Berlin, Germany; ^2^Centre for Mental Health, Bundeswehrkrankenhaus Hamburg, Hamburg, Germany

**Keywords:** COVID-19, medical staff, military, mental health, adjustment

## Abstract

**Introduction:**

The COVID-19 pandemic changed not only the working conditions but also the private conditions we live in. Health care professionals especially were confronted with multiple stressors, e.g., the risk of infection, lack of staff, and high workloads.

**Methods:**

To estimate some of the pandemic-related impacts this anonymous personnel survey was conducted in two German military hospitals (Hamburg and Berlin). This study presents a comparative analysis of the hospital staff in general vs. the psychiatric personnel (*N* = 685) at two measurement time points (MTPs) in April 2021 (*n* = 399) and December 2021 (*n* = 286). The survey contains the German version of the Covid Stress Scale (CSS) to assess the perceived level of pandemic-related stress, the Patient Health Questionnaire (German Version: PHQ-D) to screen for three major mental disorders, and the adjustment disorder—New Module (ADNM) to estimate the problems of adaptation to change.

**Results:**

The results showed a process of adaptation over the two MTPs with significant stress reduction at MTP2 in the general staff. The psychiatric staff did not report significantly higher pandemic-related symptoms. Quite the contrary, not only did the CSS show significantly lower xenophobia, traumatic stress, and compulsive checking, but the PHQ also showed lower stress symptoms and somatic symptoms at both MTPs. Also, the ADNM scores delivered evidence for a more effective adaptation process in psychiatric personnel (e.g., depressive mood, avoidance, anxiety).

**Discussion:**

The presented results must be interpreted while taking the unique situations of German military clinics into account. The supply of protective material was sufficient and there was no dramatic shortage of psychiatric staff during the pandemic. The inpatients were quite often (40%) elective treatments for trauma-related disorders, which could be discontinued in the case of a COVID-19 infection. The results of this study showed good adaptative skills among the psychiatric staff in military hospitals, which could be interpreted as a sign of good resilience. This might have led to lower stress-related symptoms during the COVID-19 pandemic.

## 1. Introduction

On the 28 January 2020, the first SARS-CoV-2 infection was detected in Germany (1). The pandemic situation progressed over the following month and medical providers were confronted with an increasing risk of devastating scenarios, which took place in neighboring countries (e.g., Italy). The outbreaks of infections had the potential to become very dynamic, exponential even, which could lead to insufficient risk control (material, isolation, etc.) (2). Not only were plans made for prioritizing strategies for patients but also regarding long-lasting facility management (e.g., personnel, material), to bear the massive workload. The pandemic became a global stressor and changed the way we work, interact and behave. But before the COVID-19 pandemic not much was known about the psycho-social aspects a pandemic has on a population in the modern day (3). The ability to cope with such changes or with the risk of infection can vary between individuals. All adaptive strategies and the outcome can be summarized under the term “resilience” (4). Especially, medical staff have been frontline actors since the beginning of the COVID-19-Pandemic and the impact on their physical and mental wellbeing has been shown in multiple studies. These workers are confronted with a very high risk of SARS-CoV2-infection (3). One of the earliest studies from Wuhan (China) was able to demonstrate impressively that healthcare professionals especially reported stress-related mental health symptoms, like depressive mood (50.4%), or anxieties (44.6%). Significant distinctions regarding profession or gender were shown in this study (5). The staff had to deal with high rates of infections, patient death, excessive workload, stress due to lack or uncertainty of information, and worries about the potential duration of the pandemic (6). These could have an impact on mental health and could be translated into an increasing shortage of already limited staff (e.g., ICU nurses), due to more frequent sick leaves.

Former pre-pandemic studies have demonstrated that work in nursing is a hard job and that nurses showed moderate to high levels of stress even before the pandemic. There were no significant differences regarding occupational stress between general ward nurses and psychiatric nurses (7, 8). The psychological stress was unrelated to age, marital status, parenthood, qualifications, or work experience (7). Not only does the job-related stress have a negative impact on the quality of a nurse’s life, but it can also affect job performance negatively (8). Another study found negative correlations between resourcefulness and stress and positive correlations with depressive symptoms (9).

In preparation for the winter of 2020, the first winter during the COVID-19 pandemic, clinics were ordered to establish in-clinic psycho-social crisis management teams, nationwide. The crisis management for the military hospitals in Berlin and Hamburg was planned and put into action by the Centre for Military Mental Health in the military hospital in Berlin and the department of psychiatry of the military hospital in Hamburg. To assess the psycho-social effects on the clinical personnel and to be able to address special needs during this pandemic, an employee survey was conceptualized in Berlin. After a positive vote in front of the ethics council of the Charité Berlin (no. EA4/04/016/21, 17.5.21), the survey was expanded to the military hospital in Hamburg, to reach more people.

These two military hospitals (Berlin and Hamburg) have similar treatment capacities, a similar broad scope of medical specialties, and a similar number of medical care providers. Both institutions are not only treating military personnel but are also involved in the regular in-patient care of civilian patients. The psychiatric departments in both hospitals have psychiatric wards, daycare clinics, and outpatient treatment centers. In contrast to other patient-treating departments within those clinics, the military psychiatric departments are treating mostly military patients with a psychotherapeutic focus.

Both hospitals were not primary COVID-19treatment facilities, identified by the state pandemic treatment plan. Instead, non-infectious patients were transferred to these facilities to receive further treatment. This led to a change in workload and staff in both clinics. COVID-19 patients were also treated on a regular basis including intensive care unit (ICU) treatment, but not specifically administered. ICU personnel in particular complained about the workload and the increase in the teaching and training of inexperienced personnel.

The aim of this study is to assess the experienced level of stress or distress in the personnel of the clinic (civilian as well as military) due to the COVID-19 pandemic and the changes in workload. We hypothesized that the impact of the pandemic situation would on the mental health of the hospital staff would decrease over time, e. g. due to vaccination campaigns and a more predictable situation (hypothesis 1).

In addition, the study was focused to explain the specific impact on the perception of stress, danger, workload, and psychological and somatic symptoms of mental health staff. In hypothesis 2, we assumed that the psychiatric staff report different specific aspects in comparison to the other clinical personnel regarding mental health symptoms, adaptation behavior, and the experience of stress.

## 2. Methods

The survey started in Berlin with a voluntary anonym employee survey in a cross-section design. We started this survey in May 2021 with a first measurement time point (MTP 1), which was followed by a second measurement time point (MTP 2) in December 2021. The data of this analysis was collected anonymously, so the participants could not be linked over the 2 measuring points.

Next to public campaigns at the clinic (e.g., posters) to advertise the survey, personnel information events were planned to motivate the hospital staff to participate in this study. The time of collection was 1 month at each MTP. To increase the probands in our study we reached out to the military hospital in Hamburg at MTP1. In Hamburg the survey was sent to all employees with a personal letter and a return envelope. We accepted surveys up to 3 months after the release of the surveys. It was possible to collect 399 surveys at MTP1 and 286 at MTP2 within those two clinics (*N* = 685).

Next to socio-demographic data the survey contained psychometric questionnaires in their German versions. Alongside other information, we asked for the specific workplace, the perceived closeness of COVID-19 patient contact, and other occupational information.

The German version of the COVID Stress Scale (CSS) was used to estimate the pandemic-related experienced level of stress. This validated 36-item scale measures the following subscales: “fear- and contamination anxiety,” “fear for economic impacts,” “xenophobia,” “compulsive checking,” “search for reassurance,” and “traumatic stress” in relation to COVID-19 (10, 11).

To screen for the most common mental health disorders and to estimate the severity, the Patient Health Questionnaire (German Version: PHQ-D) was included in this survey (12). This instrument is based on the Diagnostic and Statistical Manual of Mental Disorders 4th Revision (DSM-IV) It covers somatoforme, depressive, anxiety, eating, and alcohol dependency disorders. Severity scales are available for depression, somatic symptoms, and stress. We excluded the gynecological part of this instrument. The Cronbach’s is a = 0.88 for depression and a = 0.79 for the somatoform subscale. With a test-re-test-reliability for the depression subscale between r = 0.81–0.96 (13).

Regarding adjustment problems during the pandemic we used the Adjustment Disorder—New Module (ADNM), with the subscales “preoccupation,” “avoidance,” and “adaptive mistakes.” The internal consistency of the subscale lies between a = 0.74–0.94, the test-re-test reliability between rtt = 0.61–0.84 (14).

The sample selection was done as a two-stage cluster sample. Data were available for 10.1% of the participants at two measurement points. By means of questionnaires, psychosocial stress in the context of the COVID-19 pandemic situation was investigated by the following main variables: stress levels due to the COVID-19 pandemic, stress experience (measured by the PHQ Stress Scale), depressive symptoms (measured by the PHQ9 depression scale), adjustment problems (measured by the ADNM).

The data of 662 participants were included in this analysis, 132 from Berlin and 530 from Hamburg. 23 surveys were excluded due to a lack of provided information. With six missing values, 284 of the participants identified as male (42.9%), 369 as female (55.7%), and two as diverse. With 26 missing values and a standard deviation of 11.43, the participants were on average 37.75 years old (MIN = 17, MAX = 65). With 147 missing values, 38.7% of the participants were employed as a temporary soldier, while 16.4% reported being a professional soldier. Another 17% were civilian employees, 1.6% were civil servants, and 2.6% were other civilian employees. With 173 missing values, 40.4% of the participants worked in inpatient or outpatient care as medical staff and 14.3% in nursing. We found that 11.9% were employed in administrative roles, while 2.6% were employed in other therapy services (e.g., physiotherapy), 2.3% in support services, and 0.8% in the counseling of patients and staff.

Statistical analyses were run with IBM SPSS Statistics 26.

## 3. Results

### 3.1. Comparison of MTP1 and MTP2

In the first step the data was analyzed regarding significant differences between the two MTPs (significantly defined as *p* < 0.05). The data of both groups was not normally distributed with respect to all scales (*p* < 0.001). A Mann–Whitney *U*-test for independent samples was used to test the main hypotheses, that adjustment problems (ADNM + Subscales) decreased from MTP1 to MTP2.

Across all scales, the test yielded significant differences concerning the respective mean adjustment problems between the two MTPs (see Table 1).

**Table 1:** Results of Mann–Whitney *U*-test with ADNM subscales as dependent variable.

Scale	*U*	*P*	*r*
Preoccupation	25,247	<0.001	0.23
Failure to adapt	25,695	<0.001	0.23
Avoidance	28544.5	<0.001	0.14
Depressive mood	27,591	<0.001	0.16
Anxiety	28,699	<0.001	0.17
Impulse disturbance	28,296	<0.001	0.17
Overall score	22929.5	<0.001	0.22

Across all scales, participants reported more severe adjustment problems on average for MTP1 than for MTP2.

Regarding the COVID-19-related stress symptoms measured by the CSS and subscales, the Mann–Whitney U test for independent samples showed a significant difference between the two MTPs concerning perceived “danger” from COVID-19 as well as “xenophobia” (see Table 2).

**Table 2:** Results of Mann–Whitney *U*-test with CSS scales as dependent variable.

Scale	*U*	*p*	*r*
Danger	28,636	*0.021*	0.09
Xenophobia	27268.5	*0.002*	0.13
Fears about economic consequences	31852.5	0.475	
Contamination fears	29493.5	0.052	
Traumatic stress symptoms about COVID-19	29939.5	0.05	
Compulsive checking and reassurance seeking	31,508	0.296	
Overall score	28,624	0.211	

On average, participants perceived more “danger” from COVID-19 over time [MTP1 (*M* = 6.76; *SD* = 4.89) < MTP2 (*M* = 7.69; *SD* = 5.1)]. The subscale “xenophobia,” however, decreased over time [MTP1 (M = 5.1; SD = 4.32) > MTP2 (*M* = 4.37; *SD* = 4.95)].

The PHQ questionnaire showed significant differences between the two MTPs regarding depressive as well as stress-associated symptoms (see Table 3).

**Table 3:** Results of Mann–Whitney *U*-test with PHQ scales as dependent variables.

Skala	*U*	*p*	*r*
Depression scale	26,703	*<0.001*	0.14
Somatization scale	26,188	0.067	
Stress scale	27450.5	*0.029*	

On average, participants reported a higher burden of depressive symptoms at MTP2 (*M* = 5.13; *SD* = 5.05) than at MTP1 (*M* = 3.84; *SD* = 4.45). Also, participants scored higher on the stress scale at MTP2 (*M* = 3.88; *SD* = 3.3) than at MTP1 (*M* = 3.55; *SD* = 3.69).

### 3.2. Analysis of the occupational impact during the pandemic for psychiatric personnel

The data of employees working in psychiatry were compared with the other clinic employees regarding COVID-19-related stress (CSS + Subscales).

Given the sample size (*n* > 500), a two-way ANOVA was performed, even when the assumption of normal distribution of the residuals in the Shapiro–Wilk test (*p* < 0.001) or the assumption of homogeneity of error variances wasn’t satisfied by Levene’s test for all scales (15) [*F*(3, 505) = 2.02; *p* = 0.11].

A 2 × 2 ANOVA with “MTP” (2/3) and “psychiatry employee” (yes/ no) as between-subject factors were performed for each subscale of the used inventories.

#### 3.2.1. COVID-19 stress scale

**Figure 1:**
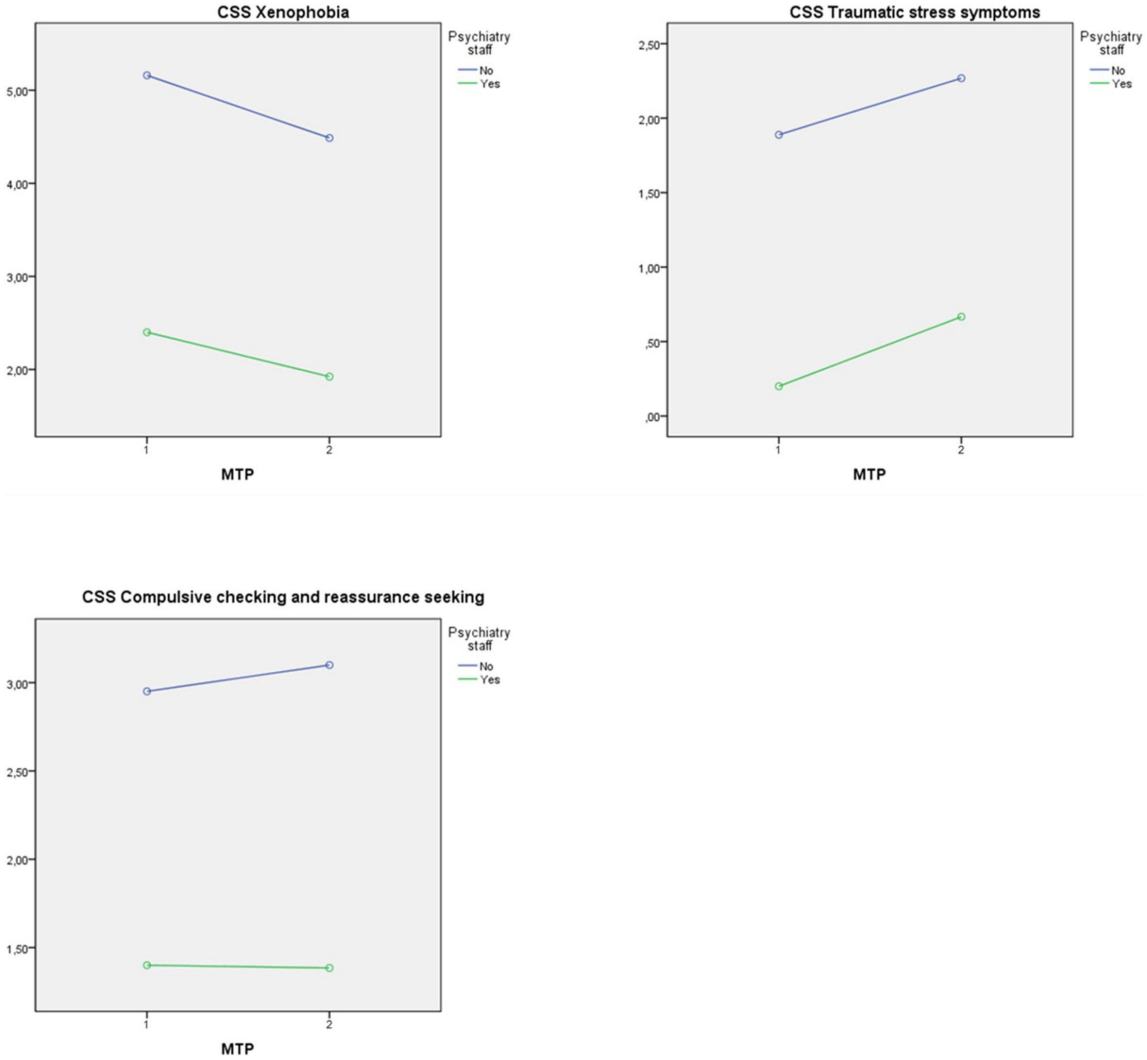
Interaction plot with dependent variables CSS-subscales.

Regarding the “xenophobia” subscale of the COVID-19 stress scale (CSS), a significant difference in psychiatric personnel was found. The main effect of the MTP as well as the interaction effect were not significant (see Table 4). The interaction plot shows that employees in psychiatry reported lower levels of “xenophobia” on average at both MTPs (see Figure 1).

**Table 4:** Results of a 2 × 2 ANOVA with CSS-subscales as dependent variable.

Subscales	*F*	*p*
**Xenophobia**		
MTP	0.213	0.323
Psychiatry	4.56	*0.017*
Interaction	0.006	0.47
**Traumatic stress symptoms**		
MTP	0.227	0.317
Psychiatry	3.433	*0.033*
Interaction	0.002	*0.481*
**Compulsive checking and reassurance seeking**		
MTP	0.005	0.467
Psychiatry	3.054	*0.041*
Interaction	0.008	0.47
**Danger**		
MTP	0.843	0.18
Psychiatry employee	1.665	0.1
Interaction	0.044	*0.417*
**Fears about economic consequences**		
MTP	0.441	0.254
Psychiatry	1.675	0.098
Interaction	0.143	*0.353*
**Contamination fears**		
MTP	0.778	0.189
Psychiatry	1.448	0.115
Interaction	0.229	*0.317*

Also, the experienced “traumatic stress” subscale revealed significant differences between the non-psychiatric and the psychiatric personnel. The other tested effects were not significant (see Table 4). The interaction plot shows that employees in psychiatry reported lower levels of “traumatic stress” symptoms on average at both MTPs (see Figure 1).

In the subscale for “compulsive checking” we found a significant main effect of working in psychiatry on participants’ experience of compulsive checking and reassurance seeking. There were no significant effects of the MTP as well as the interaction effect (see Table 4). The interaction plot shows that employees in psychiatry on average reported lower levels of “compulsive checking and reassurance seeking” at both MTPs (see Figure 1).

Neither significant main effects nor a significant interaction effect could be found on the remaining three subscales, such as participants’ experience of “danger” from COVID-19, “fears about economic consequences,” and “contamination fears” (see Table 4).

#### 3.2.2. Patient health questionnaire

**Figure 2:**
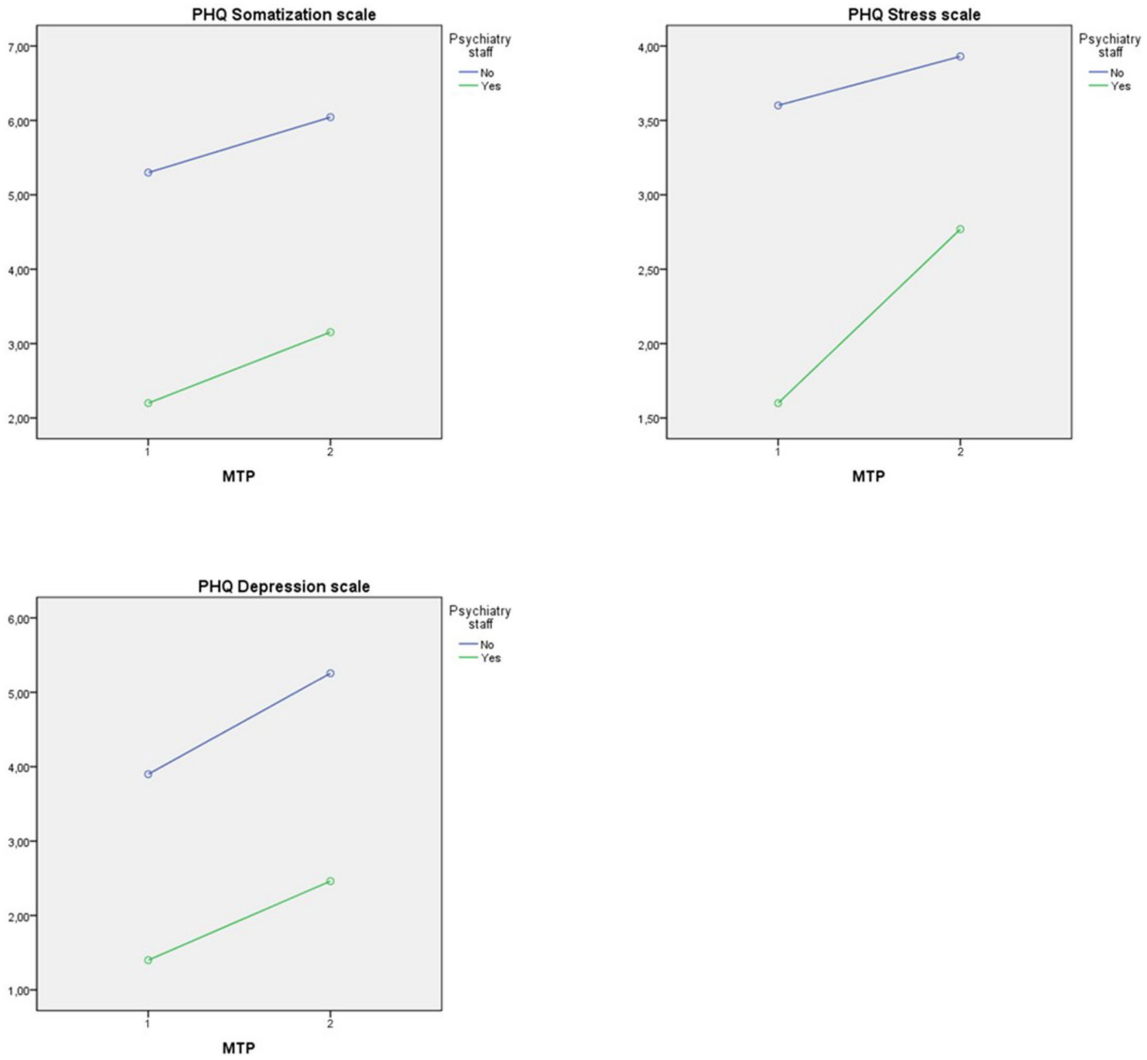
Interaction plot with dependent variables PHQ-subscales.

The three subscales of the patient health questionnaire (PHQ) were analyzed separately. The analysis for the “somatization” subscale yielded a significant main effect in psychiatric personnel (see Table 5). The staff in the department of psychiatry reported significantly lower somatic symptom distress on average at both MTPs (see Figure 2).

**Table 5:** Results of a 2 × 2 ANOVA with PHQ-subscale as dependent variable.

Subscales	*F*	*p*
**Somatization**		
MTP	0.379	0.27
Psychiatry	4.706	*0.016*
Interaction	0.006	0.47
**Stress**		
MTP	0.654	0.21
Psychiatry	2.911	*0.045*
Interaction	0.205	0.33
**Depression**		
MTP	0.899	0.172
Psychiatry	4.305	*0.02*
Interaction	0.013	0.454

Also, the scores of the PHQ “stress” subscale were significantly different in psychiatric personnel (see Table 5). The employees in psychiatry experienced lower stress symptoms on average at both MTPs (see Figure 2).

The ANOVA tests for the subscale “depressive symptoms” showed a significant difference in the psychiatric staff. The effect of the MTP and the interaction effect were not significant (see Table 5). The interaction plot shows that employees in psychiatry reported lower depressive distress on average at both MTPs (see Figure 2).

#### 3.2.3. Adjustment disorder—new module

**Figure 3:**
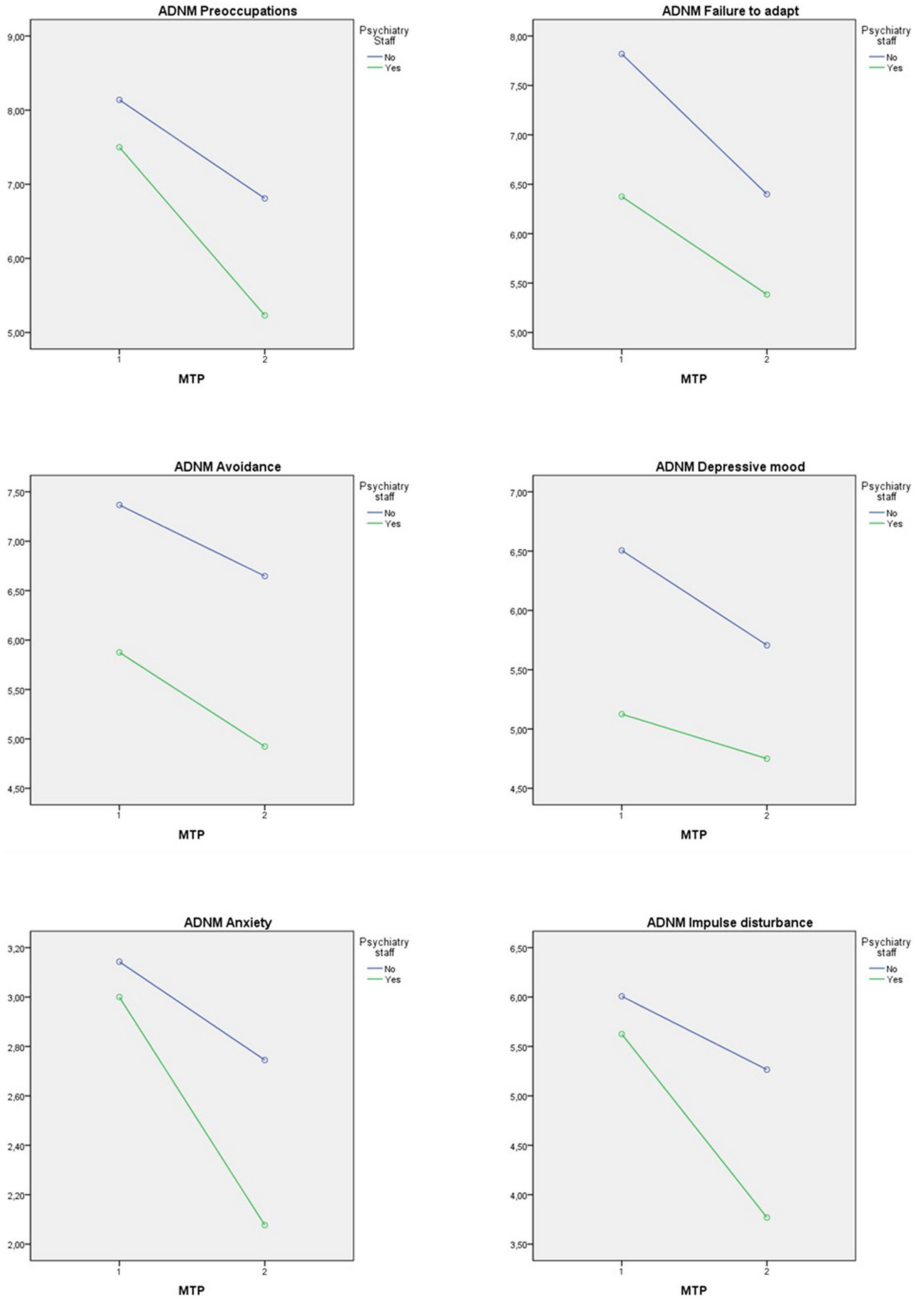
Interaction plot with dependent variables ADNM-subscales.

The six subscales of the adjustment disorder—new module (ADNM) were analyzed separately. The subscale “preoccupation” changed significantly over the two MTP, but there were no significant effects of working in psychiatry or an interaction effect (see Table 6). The interaction plot shows that both employee groups experienced more preoccupation at MTP1 than at MTP2 (see Figure 3).

**Table 6:** Results of a 2 × 2 ANOVA with ADNM subscales as dependent variable.

Subscales	*F*	*p*
**Preoccupation**		
MTP	5.667	*0.009*
Psychiatry	2.151	0.072
Interaction	0.387	0.194
**Failure to adapt**		
MTP	2.947	0.044
Psychiatry	3.067	*0.04*
Interaction	0.093	0.38
**Avoidance**		
MTP	1.245	0.133
Psychiatry	4.61	*0.02*
Interaction	0.024	0.439
**Depressive mood**		
MTP	0.979	0.162
Psychiatry	3.881	*0.025*
Interaction	0.128	0.361
**Anxiety**		
MTP	4.325	0.019
Psychiatry	1.63	0.101
Interaction	0.684	0.205
**Impulse disturbance**		
MTP	4.349	*0.019*
Psychiatry	2.274	0.066
Interaction	0.799	0.186

Also, the subscale “failure to adapt” showed significant main effects on participants’ experience (see Table 6). The interaction effect was not significant. The interaction diagram shows that both employee groups experienced more failure to adapt at MTP1 than at MTP2 and that psychiatry employees showed less failure to adapt at both MTPs (see Figure 3).

Significant differences for the personnel in psychiatry were also found in the “avoidance” subscale. The effects of MTP and interaction were not significant (see Table 6). The interaction diagram shows that employees in psychiatry on average reported less avoidance at both MTPs (see Figure 3).

The “depressive mood” was significant for the psychiatric staff, with no significant main effect of the MTP or interaction effect (see Table 6). The interaction plot shows that employees in psychiatry on average reported less depressive mood at both MTPs (see Figure 3).

The “anxiety” subscale yielded a significant main effect over the two MTPs, but no such effects for working in psychiatry or interaction effect (see Table 6). The interaction diagram shows that both employee groups scored higher anxiety scores at MTP1 than at MTP2 (see Figure 3).

The results of the subscale “impulse disturbance” over the two MTPs differed significantly. The main effect of working in psychiatry and the interaction effect were not significant (see Table 6). The interaction diagram shows that both employee groups experienced more strain in the area of impulse disturbance at MTP1 than at MTP2 (see Figure 3).

## 4. Discussion

According to our first hypothesis, we assumed that stress levels would decrease from MTP1 to MTP2. In fact, the opposite was the case. The perceived level of stress in our total sample increased over time. In addition to that, depressive symptoms increased between MTP1 and MTP2, while anxiety decreased over time.

One possible explanation could be, that the hospital staff developed some sort of exhaustion over the duration of the pandemic. This aspect might have been intensified after the assumption of a quick normalization in patient care. The lack of personnel in times of high viral infection rates with COVID-19 in the population may also have contributed to this increase in stress. The combination of prolonged intensive workloads with multiple uncertainties about the situation may have led to a dishabituation of the hypothalamic–pituitary–adrenal (HPA) system, resulting in lower cortisol levels, which have a major impact on mood regulations (16).

Furthermore, a cross-sectional study showed significant mental health impairment among staff in UK COVID-19-treating hospitals of the National Health System Trust, which was attributed to increased exposure to moral injury (17). Maunder et al. showed similar results in their study about psycho-social stress in nursing staff, that over time the signs for emotional exhaustion increased and peaked after 6 months (18).

A military hospital sample using a burnout inventory showed low levels of burnout syndrome, with a peak during the first wave of the pandemic, with a decline at the end of the first wave, and then a continuous low level of relevant symptoms. Profession-specific differences were also found. It is remarkable that nursing personnel were less frequently affected than young doctors. This study also proved that female staff reported burnout syndromes less often than male staff (19).

In contrast, a study on a German university, which investigated gender differences in hospital staff regarding anger showed a higher score of anger in women, with direct contact with COVID-19-affected patients having no influence on the perception of anger (20). Perceived social support as well as a high sense of coherence have a decreasing impact on mental pathology and seem to be very valuable for the mental health status of healthcare workers during the COVID-19 pandemic, as demonstrated by a large evaluation study in German hospitals (21).

Regarding the subgroup-analysis, we assumed that mental healthcare personnel would report different aspects of stress or mental health symptoms. The staff of our sample reported a significantly higher perception of danger at MTP2, while the intensity of xenophobia decreased.

But we did not find any differences in the group comparison between psychiatric and non-psychiatric staff in the experience of danger and economic consequences in this study.

Regarding the perceived danger it seems plausible that all healthcare professionals in the two military hospitals had access to somewhat comparable information about the ongoing pandemic. This includes knowledge about the virus, treatment options, and the proper way to protect the staff. In addition to that, podcasts and videos of the commanders were made available below the usual threshold for this purpose. In the hospitals of our study, media campaigns on COVID-19 as well as on stress and the possibilities of influencing resilience were also carried out, which are still accessible on public media portals today.

It could be assumed that the hospital staff had a good awareness about the fact, that very problematic, life-threatening infections were rather rare cases, but that the vast majority of infections showed mild symptoms and had a good outcome. Most of the personnel deployed in the German Armed Forces hospitals were military personnel or reserve military servicemembers with a reasonably good health status, which is necessary and mandatory for military service. Complicated courses in case of a COVID-19 infection were rather unlikely for this group. This became especially true after the vaccination against COVID-19 started in the beginning of 2021. Also, other studies were able to demonstrate similar decreasing effects on the fear of contracting COVID-19 after vaccination. On the other side, an excessive demand for personal protective equipment masks remained (22). There were no documented shortages in excess of protective equipment, which could have resulted in an additional stressor.

Regarding the economic situation, we must constate, though, that there was a nationwide need for hospital staff at the time our survey was conducted. The health care system in Germany had a high demand for specialized clinical staff (e.g., ICU nurses, doctors). The job situation was therefore not to be described as uncertain. Rather the contrary, financial bonuses were paid out to the personnel treating COVID-19 in Germany, in order to show appreciation for their performance, but also to retain personnel. It must be taken into account that more than 2/3 of the personnel in our sample were soldiers, with very stable working conditions and fixed contracts or lifelong careers in the military.

There were some significant differences in the subgroup analysis (psychiatric staff compared to the non-psychiatric staff). Traumatic stress was reported less frequently by staff of the department of psychiatry at both MTPs. Also, significantly lower levels of compulsive checking and reassurance seeking of the psychiatric caregivers were reported at both measurement points.

From the authors’ point of view, this can be explained by the fact that only necessary inpatient treatments were carried out. Elective inpatient admissions were linked to regular COVID-19 testing or, in the later course of the pandemic, to vaccination statuses. In case of COVID-19-infections, patients only remained in isolation as inpatients if a psychiatric treatment was urgently needed or if the infection led to significant symptoms which required special medical treatment. Many psychiatric inpatients are electively treated with a psychotraumatological emphasis, which made it common to discharge patients that have tested positive for COVID-19 for isolation at home and to readmit them later for a continuation of the treatment after negative test detection. The psychiatric staff were thereby not confronted by severely sick or even dying patients. As shown in other studies (23), the procedure to release patients with COVID-19-infection causes potential problems in patient care due to the disrupted treatment process and the limited possibilities of group therapies but seems to have protective effects on the stress perception of the psychiatric staff. In addition to that, treatment was also provided by the psychiatric departments of the Bundeswehr hospitals via web-based therapy, which sparked a “rethinking” of new approaches in treatment and digital health. Similar developments have also been seen in the civilian healthcare system (24). In fact, web-based treatment was relatively common among the patients in military hospitals, as the psychiatric clinics treat mainly stress-reactive and depressive disorders, which seem to be adequately addressable via online contact. Civilian general psychiatry on the other hand is mainly confronted with more severe disorders (e.g., schizophrenia, dementia) and patients with low social economic resources (e.g., no computer/smartphone, homelessness), which makes web-based treatment not as generally applicable, even though psychiatric staff also partly assessed the possibility of teletherapy as a positive consequence of the pandemic (25).

The significantly lower level of compulsive checking and reassurance seeking of the psychiatric caregivers at both measurement points can also be explained by the situations described above, which made the pandemic a rather manageable situation with clear processes in case of infections and a low risk for own infection.

In addition to the lower COVID-19-associated stress experience, fewer depressive and somatic symptoms were reported in the PHQ among psychiatric staff. Here, too, the authors assume that this was possibly due to the good risk management of the situation with a feeling of controllability, but also clear processes with a good level of reliability and predictability. The significantly lower stress experience in the CSS inventory of the psychiatric staff group was also shown on the stress scale of the PHQ at both MTPs.

Additional physical effort was only justified by the mandatory consistent wearing of the FFP2 masks. There was no increased demand in physical patient care. The psychiatric staff was sometimes ordered to support some somatic wards of infectiology or internal medicine, but only for a very limited period of time. However, there were COVID-19-infections among the psychiatric staff which lead to staff shortages during the pandemic, there were no documented cases with serious health consequences and the situation still remained manageable.

There was a significantly lower level of xenophobia among the psychiatric staff at MTP1. From the authors’ point of view, this may be related to the fact that psychiatric staff more often having higher levels of social and intercultural competencies, and that almost exclusively German military personnel were treated as inpatients. It could also be suspected that there is a selective effect because the proportion of patients with a different cultural background in the psychiatric field is very high and it seems likely that only personnel who does not feel xenophobic tendencies in the first place choose to work in the field of psychiatry.

The results of the ADNM seem to support the results presented for the PHQ and the CSS. Although, both, psychiatric and non-psychiatric staff, reported higher levels of preoccupation at the beginning of the pandemic. The results show that in the early stage of the pandemic, the general symptomatology was more likely to lean toward an adjustment disorder. The initial situation led to significant preoccupation, which was shown in the total sample by the increased preoccupation scale of the ADNM. In the further course of the pandemic, this was followed by depressive symptoms in the sense of exhaustion, fatigue, and loss of strength, as shown in the results of the PHQ-9 in our sample (18).

But the preoccupation as well as maladjustment, avoidance and depressiveness were significantly lower among psychiatric staff than among the non-psychiatric hospital staff.

The ADMN shows also, that the anxiety and impaired impulse control decreased over time. This could be explained by the better expectability of the pandemic situation and a better risk perception over the course of the pandemic. There were no significant group differences detectable. However, the majority of the general hospital staff were not involved in direct COVID-19-associated care. COVID-19-associated care in both hospitals was mainly provided by the departments of internal medicine, infectious diseases, anesthesia, and emergency and rescue medicine. However, all clinical departments were available for consultation and liaison services. Departments such as physiotherapy, laboratory medicine, microbiology as well as radiology were more involved in diagnostic and therapeutic processes than other departments, such as neurosurgery, visceral surgery, ophthalmology, or urology. Thus, the authors assume that there were comparable challenges and consequences for staff in psychiatry as well as for the rest of the hospital staff. It seems possible, however, that there was even a decrease in workload in military psychiatry, as COVID-19-infected patients could be discharged, as described above.

While in the general sample the levels of preoccupation, anxiety, and impulse control were similar, the stresses were less likely to lead to symptoms in the domain of depressed mood, avoidance behavior, and disturbances in adjustment, both cross-sectionally and longitudinally.

Interestingly, samples of civilian psychiatric hospitals showed higher levels of stress experience and negative impact on well-being (26), which is why aspects such as the better health status of military personnel, the good staffing and equipment, as well as military unit cohesion and military leadership, are factors that could increase resilience. It must also be considered that the COVID-19-associated burdens and consequences in the hospital systems varied not only nationally but also regionally. However, the very controllable situation in the two hospitals studied is not comparable with the situation of emergency reserve hospitals, rather classic field hospitals, where the functions of large hospitals were taken over to relieve them in critical situations, for example in the United States (27).

In summary, the comparison of the COVID-19-pandemic-related impact on mental health, adaptation behavior, and the experience of stress in hospital staff showed partial group differences in the aspect of a lower stress load and lower mental and somatic symptoms for the psychiatric staff, but comparable risk perception and fears, e.g., of infection. Furthermore, the stress perception and depressiveness in the total sample increased over the MTPs. Only by assessing the effects of a scenario like this pandemic it might be possible to understand underlying dynamics in clinical teams, which promote stress experience, mental health, mental health symptoms, or adaptive strategies. Thereby it might be possible to counteract to sustain a productive work environment and to protect persons at risk for negative consequences (e.g., work fatigue, depression).

The manageability of the pandemic situation in many hospitals in Germany, but especially in the Bundeswehr hospitals, may have contributed to these results. Hierarchical structures in the military and a military-specific unit cohesion also might have positive effects on coping strategies and resilience (28).

In addition to that, primary preventive strategies should be advertised on a low threshold level and there should be an emphasis on positive appreciation for the clinical staff.

*Ad hoc* research as a health monitoring of hospital staff makes sense and should also be carried out in a similar way in civilian hospitals. With this project, real-time health monitoring should identify mental problems more quickly to adopt operational response opportunities for managers and employers. Based on our findings, measures to support hospital staff were introduced and guided in both hospitals. However, from the authors’ point of view, the comparability of parameters like stress perception with civilian hospital staff was limited. Due to the rapid activation of the military reserve of medical soldiers and in contrast to the staffing situation in civilian hospitals, staff shortages in the military hospitals were well compensated. Nevertheless, in pandemic situations, regular monitoring of the impact on the mental health of civilian hospital staff can be also recommended. However, it must be noted, that this is certainly easier for hospitals (e.g., university clinics or military hospitals) with their own research facilities than for regular basic treatment facilities.

In a follow-up study, we plan to assess the effects of working closely with COVID-19 patients in a comparative analysis within the same sample of clinical staff. Thereby we hope to get a better understanding of the role of the COVID-19-specific stress within those general effects presented in this study.

## 5. Limitations

This study relies only on self-reported data that was collected via voluntary and anonymous questionnaires. An over-reporting and a response bias are possible. The validity of the reported data could not be verified.

Since missing values were handled using pairwise deletion in this study, the sample size varies over the different analyses reported in this article.

The data analyzed in this article was collected from staff in military hospitals in Berlin and Hamburg. Both clinics have psychiatric wards for inpatient treatment with a strong emphasis on post-traumatic stress disorders. Most of these patients are active military personnel, and only the psychiatric clinic in Berlin treats a few civilian inpatients (*ca.* 17%). The job-related demands for the staff differ from those in general psychiatry. The degree of violence and aggressive behavior is rather low and the patient clientele is mostly able to understand and follow rules (e.g., isolation). Thus, drawing conclusions for staff members of general psychiatric wards is limited and could be the subject of another study.

Due to the elective nature of the therapeutic offers, patients who had tested positive for COVID-19 could in almost all cases be discharged. Hence, the contact with COVID-19 in the work environment was very limited for psychiatric staff.

The potential role of resilience, military leadership, and military unit cohesion was discussed, but no instrument was used to detect or estimate those aspects. But not only can military leadership positively interfere with the presented experiences of occupational stress, but toxic leadership might also lead to opposing effects and result in additional psychological symptom burdens for the staff.

The data in this study were collected at different measurement time points within 1 year. While MTP 1 data was collected in the summer of 2021, MTP 2 data was collected in the winter of 2021. It has to be taken into account that COVID-19 infection rates, as well as the extent of political measures to contain the pandemic (e.g., isolations, curfew), increased during the winter months. Against this backdrop, the variety of confounding variables must be considered when discussing possible explanations for the results.

## Data availability statement

The raw data supporting the conclusions of this article will be made available by the authors, without undue reservation.

## Ethics statement

The studies involving human participants were reviewed and approved by the Ethics Committee of Charité University Berlin, Germany. Written informed consent for participation was not required for this study in accordance with the national legislation and the institutional requirements.

## Author contributions

G-DW and CH: study funding, ethics, manuscript conception, and draft. G-DW, CH, and JM: study planning and sample recruitment. NS, CH, and G-DW: analysis and statistics. G-DW, CH, NS, JM, HH, UW, and PZ: review and corrections. All authors contributed to the article and approved the submitted version.

## Funding

This study was funded and approved by the Federal Ministry of Defence (grant number: 35K4-S-32 2023).

## Conflict of interest

The authors declare that the research was conducted in the absence of any commercial or financial relationships that could be construed as a potential conflict of interest.

## Publisher’s note

All claims expressed in this article are solely those of the authors and do not necessarily represent those of their affiliated organizations, or those of the publisher, the editors and the reviewers. Any product that may be evaluated in this article, or claim that may be made by its manufacturer, is not guaranteed or endorsed by the publisher.
